# Megakaryocytes, erythropoietic and granulopoietic cells express CAL2 antibody in myeloproliferative neoplasms carrying CALR gene mutations

**DOI:** 10.1111/iep.12375

**Published:** 2020-09-14

**Authors:** Hebah Ali, Ignazio Puccio, Ayse U. Akarca, Roshanak Bob, Sabine Pomplun, Wai Keong Wong, Rajeev Gupta, Mallika Sekhar, Jonathan Lambert, Hytham Al‐Masri, Harald Stein, Teresa Marafioti

**Affiliations:** ^1^ Haematological Malignancy Diagnostic Service Leeds UK; ^2^ University of Leeds Leeds UK; ^3^ Department of Histopathology University College London London UK; ^4^ Reference and Consultation Center for Lymphoma and Haematopathology Pathodiagnostik Berlin Berlin Germany; ^5^ Department of Cellular Pathology University College Hospital London UK; ^6^ Department of Haematology University College Hospital London UK; ^7^ Hematogenix Laboratory Services Chicago IL USA

**Keywords:** CAL2, CALR, essential thrombocythaemia, mutated calreticulin, myelofibrosis, myeloproliferative neoplasms

## Abstract

Testing for the CALR mutation is included in the updated WHO criteria for essential thrombocythaemia (ET) and primary myelofibrosis (PMF). We report on the application of the CAL2 monoclonal antibody, raised against the mutated CALR gene to myeloid cases. The immunostain was used on 116 acute myeloid leukaemias (AML) and 66 myeloproliferative neoplasms (MPN) or myelodysplastic syndromes/myeloproliferative neoplasms (MDS/MPN). None of AML cases was stained by the CAL2 antibody, while 20/66 MPNs and MDS/MPNs appeared positive. Fourteen of the latter cases were studied by molecular techniques, and all showed aberrations of the CALR gene. In addition, CAL2 positivity was found in some small‐sized elements besides megakaryocytes. By double staining, these elements corresponded to small megakaryocytes as well as both erythroid and myeloid precursors. This finding suggests possible occurrence of CALR gene abnormalities in a stem cell.

## 1 INTRODUCTION

The discovery of mutated Calreticulin (CALR) in myeloproliferative neoplasms (MPN) has provided proof of clonality, diagnostic importance and influence on prognosis of this pathology. The identification of this MPN‐associated driver mutation—currently based on molecular assays—is represented as a major diagnostic criterion for essential thrombocythaemia (ET), prefibrotic myelofibrosis and primary myelofibrosis (PMF) in the updated World Health Organization (WHO) 2008 classification.^1^ Recently, Vannucchi et al^2^ and Stein et al^3^ raided polyclonal and monoclonal antibodies against mutated CALR to be used in the routinely processed bone marrow paraffin sections as a complementary assay to detect the mutant CALR protein in patients with MPNs. In the present study, we validated by immunohistochemistry the diagnostic usefulness of the monoclonal CAL2 antibody. Cases of acute myeloid leukaemia (AML) and myelodysplastic/myeloproliferative neoplasms (MDS/MPN) have been also investigated to assess the specificity of CAL2 antibody. For this purpose, the result of the CAL2 immunostaining was compared with the result of molecular assays. Additionally, we investigated by double staining whether expression of mutated CALR can also be demonstrated on cells of the erythroid and myeloid lineage.

## 2 MATERIAL AND METHODS

### 2.1 Tissue samples

One hundred and eighty‐two bone marrow biopsies from patients with myeloid neoplasms: series of MPNs (n = 66) and a control group of acute myeloid leukaemia (AML) (n = 116) were retrieved from the files of the Department of Histopathology at University College London Hospitals. The cases were diagnosed by expert haematopathologists and haematologists at University College London Hospitals following the criteria of the 2008 WHO classification.^1^ Approval for this study was obtained from the National Research Ethics Service, Research Ethics Committee 4 (REC Reference number 09/H0715/64).

### 2.2 Immunohistochemistry

The bone marrow biopsies were fixed in 10% neutral‐buffered formalin, decalcified for 6 hours using Gooding‐Stewart solution, processed and embedded in paraffin. Immunostaining using the newly developed anti‐human CAL2 monoclonal mouse antibody^3^ was performed on bone marrow biopsies tissue sections using the Roche‐Ventana BenchMark ULTRA autostainer (Ventana Medical Systems).

The CAL2 antibody was assessed under different conditions (ie dilution and antigen retrieval protocols), and the chosen dilution, which showed selective background‐free reaction, was 1:10. Counterstaining was performed using haematoxylin and bluing reagent from Ventana/Roche. Slides were mounted with cover slips and air‐dried.

### 2.3 Double immunostaining

Double immuno‐enzymatic labelling of biopsies’ sections following the described pretreatment was carried out. Primary antibodies were incubated for 30 minutes at room temperature, and a diaminobenzidine (DAB) substrate (DakoCytomation) was then used for the detection of antibody binding. Sections were then incubated for 30 minutes with the second antibody. The second reaction was detected by means of a Vector Blue Alkaline Phosphatase or a Fast Red. The sections were washed in tap water and mounted in aquamount (Merck). Double immunostaining was carried out to analyse the expression of CD71, myeloperoxidase, CD61 and GATA‐1 in combination with CAL2 antibody.

The cases were reviewed by an expert haematopathologist (TM), a co‐author of this paper.

### 2.4 Molecular assay

For some cases included in this study, molecular analysis of CARL gene and a set of other genes including JAK2, MPL, BCR/ABL1 and KIT were performed using a combination of fragment analysis and sequencing. The assays used are ipsogen JAK2 MutaSearch kit, Qiagen Ipsogen MPL W515KL MutaScreen assay, GenMark BCR‐ABL1 (p190, p210 and p230) screening kit and Plentiplex Mastocytosis KIT D816V assay. DNA was isolated from peripheral blood or bone marrow aspirate. DNA was amplified using forward and reverse primers spanning exon 9 of the CALR gene with the forward primer fluorescently labelled. CALR F: (5′ FAM‐GGCAAGGCCCTGAGGTGT 3′) and CALR R: (5′‐GGCCTCAGTCCAGCCCTG 3′). The conditions were as follows: (1×) 95.0°C for 15 min; (10×) 94.0°C for 15 seconds, 55.0°C for 15 seconds, 72.0°C for 30 seconds; (20×) 89.0°C for 15 seconds, 55.0°C for 15 seconds, 72.0°C for 30 seconds; and (1×) 72.0°C for 10 min. PCR products were analysed by capillary gel electrophoresis. 100 ng of gDNA extracted using QIAamp DNA Blood Mini Kit (Qiagen) was used in each assay.

## 3 RESULTS

CAL2 immunostaining was evaluated in a total of one hundred and eighty‐two bone marrow biopsies from patients with myeloid neoplasms (AML n = 116, MPNs n = 66) (Table 1). Positivity was observed in twenty out of sixty‐six biopsies from patients with MPNs that included the following: ET n = 14, chronic MPN, with MF n = 3; MDS/MPN, unclassifiable n = 2; MF n = 1 (Table 2). In 14 of the 20 CAL2‐positive cases, PCR or sequencing was performed and results showed CALR molecular aberrations (either mutation or deletion etc) (Table 2).

**Table 1:** CAL2 immunohistochemistry staining results in bone marrow biopsies obtained from patients with myeloproliferative neoplasms and acute myeloid leukaemia

Diagnosis	Total number	CAL2 staining (positive/total)
AML	116	0/116
MPN	66	20^a^/66

Abbreviations: AML, acute myeloid leukaemia; MPN, myeloproliferative neoplasms.

^a^ The 20 CAL2‐positive cases are detailed in Table 2.

**Table 2:** Clinical, pathological and molecular data of myeloproliferative cases with CAL2‐positive immunostaining

ID	P/R	Diagnosis	Molecular CALR aberrations/others	CAL2 IHC
ID1	P	ET	2‐bp insertion in exon 9 and a c.149_1154del and ins TCCTTGTC resulting in predicted protein change p. (Glu383Aspfs*48)	+
ID2	P (2012)	ET	Not performed	+
ID2.1	R **(**2014)	Chronic MPN with MF	5‐bp insertion in exon 9. The mutation load was approx. 47%. The mutation c.1154_1155ins TTGTC resulting in predicted protein change p. (Lys385Asnfs*47)	+
ID3	P	ET	Not performed	+
ID4	P	ET	Not performed	+
ID5	P	ET	Not performed	+
ID6	P	MDS/MPN, unclassifiable	CALR exon 9 mutation	+
ID7	P	ET	Not performed	+
ID8	P	MDS/ MPN, unclassifiable	52‐bp deletion; the mutation load was 45%. The mutation c.1099_1150del resulting in predicted protein change p. (Leu367Thrfs*46)	+
ID9	P	ET	Not performed	+
ID10	P	ET	52‐bp deletion; the mutation load was 32%. The mutation c.1099_1150del resulting in predicted protein change p. (Leu367Thrfs*46)	+
ID11	P	Chronic MPN with MF	Not performed	+
ID12	P	ET/early‐stage MF	CALR+, BCR/ABL (−), JAK2 (−), MPL (−), KIT (−)	+
ID13	P	Chronic MPN with MF	CALR+, BCR/ABL (−), JAK2 (−), MPL (−), KIT (−)	+
ID14	P	ET/early‐stage MF	CALR+, BCR/ABL (−), JAK2 (−), MPL (−), KIT (−)	+
ID15	P	ET/early‐stage MF	CALR+, BCR/ABL (−), JAK2 (−), MPL (−), KIT (−)	+
ID16	P	ET	CALR+, BCR/ABL (−), JAK2 (−), MPL (−), KIT (−)	+
ID17	P	ET	CALR+, BCR/ABL (−), JAK2 (−), MPL (−), KIT (−)	+
ID18	P	Early‐stage MF	CALR+, BCR/ABL (−), JAK2 (−), MPL (−), KIT (−)	+
ID19	P	MF	CALR+, BCR/ABL (−), JAK2 (−), MPL (−), KIT (−)	+
ID20	P	ET	CALR+, BCR/ABL (−), JAK2 (−), MPL (−), KIT (−)	+

Abbreviations: ET, essential thrombocythaemia; MDS/MPN, myelodysplastic/myeloproliferative neoplasms; MF, myelofibrosis; MPN, myeloproliferative neoplasm; P, primary diagnosis; R, relapse.

**Figure 1:**
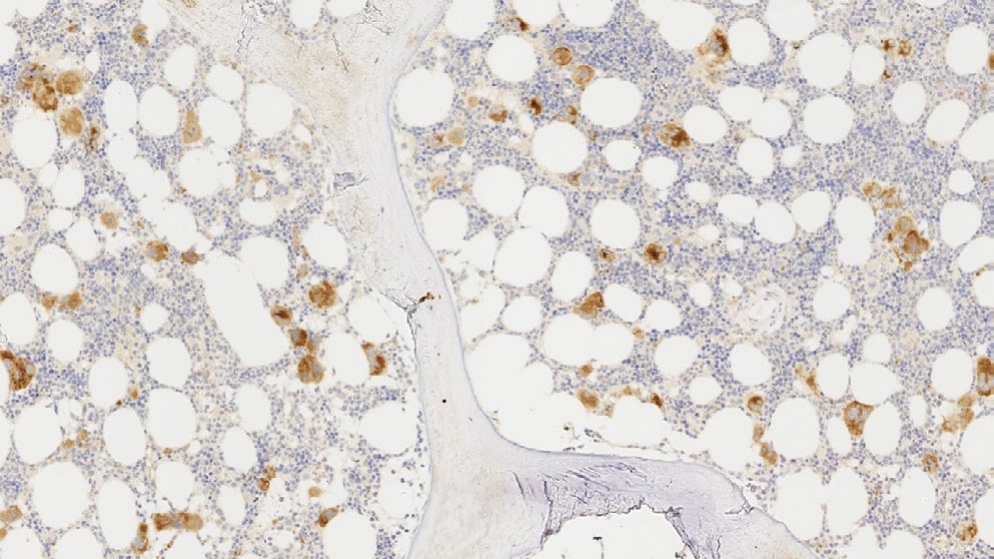
Immunostaining with CAL2: The megakaryocytes show strong expression in bone marrow biopsies from an essential thrombocythaemia patient. Magnification: ×4

**Figure 2:**
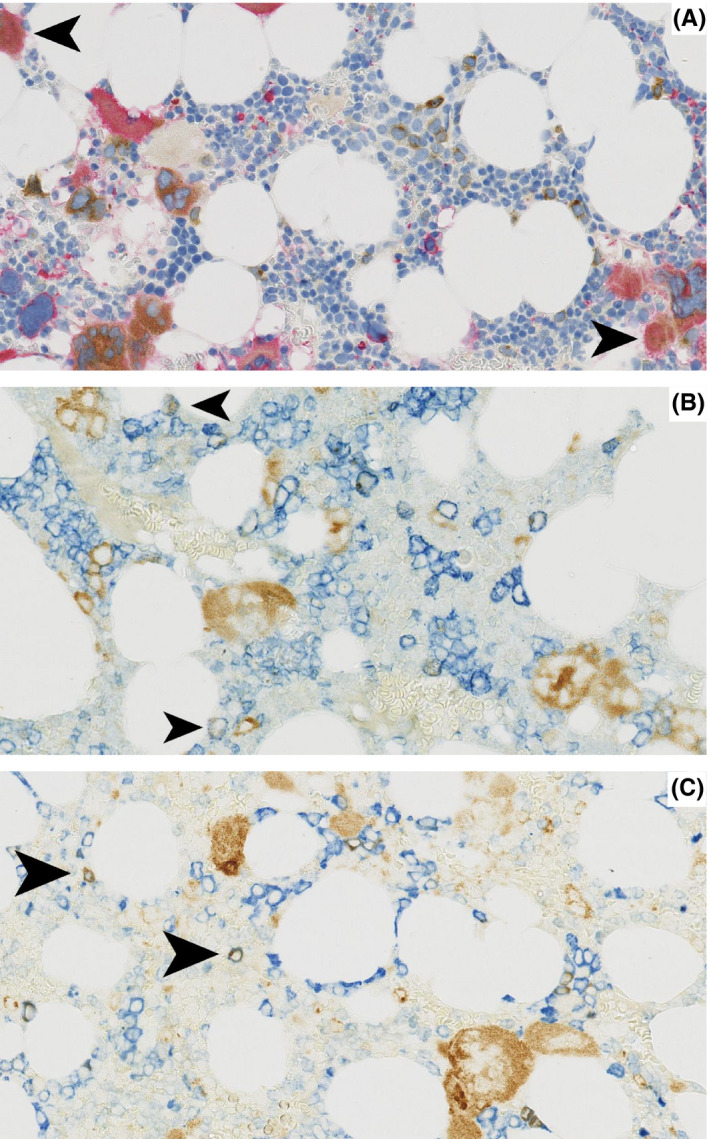
Double immunostaining with CAL2 and CD61, CD71 and MPO: CAL2 (brown cytoplasmic) is expressed in megakaryocytes as well as smaller cells: (A) Micro‐megakaryocytes: CD61 red cytoplasmic, (B) Erythroid precursors: CD71 blue cytoplasmic and (C) Myeloid elements: myeloperoxidase blue cytoplasmic. Magnification: x40

Mutated CALR expressions shown with CAL2 were mostly restricted to megakaryocytes, principally labelling the cytoplasm and displaying a granular staining pattern (Figure 1). Even some small CAL2‐positive cells proved to be positive for CD61 thus identifying these cells as small megakaryocytes (Figure 2). However, occasional smaller cells with round nuclei were stained by CAL2 antibody and antibodies to myeloperoxidase or to CD71, showing that a few granulopoietic (myeloperoxidase positive) and erythropoietic (CD71 positive) cells express mutated CARL2.

**Figure 3:**
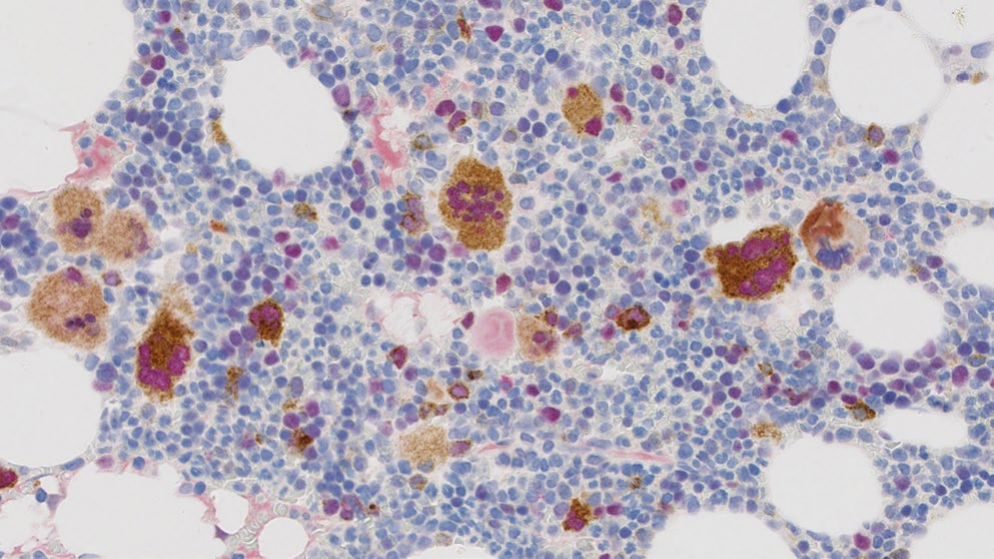
Double immunostaining with CAL2 and GATA‐1: The megakaryocytes show double expression of CAL2 (brown cytoplasmic) and GATA‐1 (red nuclear). Magnification: ×40

None of the acute myeloid leukaemia biopsies showed a positive staining with the CAL2. Double staining showed the CAL2‐positive megakaryocytes in essential thrombocythaemia patient to co‐express GATA‐1 (Figure 3).

## 4 DISCUSSION

Mutations in CALR have been discovered in 50%‐80% of JAK2 and MPL wild‐type patients with Philadelphia‐negative MPNs.^4^ CALR mutation as a gain‐of‐function mutation exhibits MPL‐dependent oncogenic property. Mutant CALR interacts with MPL and activates downstream signalling molecules that induce the JAK2 activation and subsequent cytokine‐independent growth. However, the precise molecular mechanism via which mutant CALR interacts and subsequently activates MPL is still not clear.^1^ The recent revision of WHO criteria for ET and PMF includes testing for the CALR mutation.^1^ Currently, molecular methods mainly by PCR are the standard for detection of these mutations. Unlike JAK2 and MPL point mutations, CALR mutations are highly heterogeneous, with several types of insertions or deletions, all located in exon 9.^5^ Because of this high heterogeneity, the molecular assays are complicated. In addition, their performance might be time‐consuming and technically or financially not feasible to many routine diagnostics laboratories. There is therefore a need for a simpler, faster cost‐effective method. Recently, two groups worked on developing both polyclonal and monoclonal immunohistochemical stains to detect mutant CALR in bone marrow biopsies.^2^, ^3^

In our study, we confirmed the usefulness of the CAL2 monoclonal antibody in successfully detecting mutant CALR in bone marrow biopsies.

We showed that the immune reactivity of CAL2 was absolutely restricted to the presence of CALR mutations, which were seen only in ET and MDS/MPN biopsies, but not in AML biopsies. There was 100% concordance in biopsy specimens with the concomitant molecular results. Our findings support the results of a previous analysis performed by Stein et al demonstrating the diagnostic utility of the CAL2 antibody^3^ and are comparable to four following studies.^6^, ^7^, ^8^, ^9^

Moreover, we were able to explore the lineage of few smaller cells expressing mutated CALR described in some of those studies. Interestingly, Vannucchi et al^2^ observed modest labelling by a polyclonal antibody in myeloid and erythroid cells. However, the lineage of similar cells could not be clarified in the first study assessing the monoclonal CAL2 antibody.^3^ The results from subsequent publications were diverse. In one 2016 study, the nine positive cases show staining in the majority of megakaryocytes with little or no staining in any other cell types.^6^ On the other hand, Nomani et al^7^ in the same year noted staining of small mononuclear in CALR mutant cases. By performing double immunofluorescence staining, they proposed that the small cells appeared to be myeloid cells or blasts. Recently, Bonifacio et al^8^ published unifying results describing two different patterns of CAL2‐positive staining. Pattern A is characterized by staining of almost only megakaryocytes. In contrast, there is staining of megakaryocytes and small elements at least partially being myeloid precursors in pattern B.

In our study, we applied double‐staining technique and confirmed that a subpopulation of granulopoietic and erythropoietic cells express mutated CALR as demonstrated with the CAL2 antibody in cases of MPNs. This supports the suggestion by Nanglia et al^10^ that the CALR mutations occur in a multipotent progenitor capable of generating both myeloid and erythroid progeny with preferential expansion of megakaryocytic cell lineage as a result of CALR mutation in an immature hematopoietic stem cell.

Additionally using double staining, we detected co‐expression of GATA‐1 in ET biopsies. This is consistent with the finding of Rinaldi et al^11^ and has relevance for the understanding of pathogenetic mechanisms associated with CALR mutations. Although at present, no direct correlation between GATA1 expression and CALR mutations is found, Brown et al^12^ observed a significant upregulation of CALR mRNA in MPN cases with high GATA‐1 and this could be relevant to the double immunohistochemistry staining in our study. This correlation should be confirmed in wider and independent series.

## 5 CONCLUSION

In conclusion, immunohistochemistry is readily available in the majority of diagnostic laboratories and the detection of CALR mutations by the CAL2 antibody represents a valuable supplement to traditional mutation testing and can help to facilitate the timely, appropriate selection and treatment of patients with myeloproliferative neoplasms with targeted therapies.

## ETHICAL APPROVAL

Approval for this study was obtained from the National Research Ethics Service, Research Ethics Committee 4 (REC Reference number 09/H0715/64).

## CONFLICT OF INTEREST

The authors have no conflict of interests.

## AUTHOR CONTRIBUTIONS

TM and HS conceived the idea and designed the study. TM, MS and JL selected the clinical cases for inclusion. PI and AUA performed immunostaining and collate the data. TM reviewed and interpreted the staining. TM and HA compiled the results and created the figures. HM, WKW, RG, MS and JL provided a substantial number of clinical cases. WKW, RG and SP involved in the diagnostic analysis of the cases. HA and TM wrote the manuscript.

Uncited reference
